# Ability of university-level education to prevent age-related decline in emotional intelligence

**DOI:** 10.3389/fnagi.2014.00037

**Published:** 2014-03-11

**Authors:** Rosario Cabello, Beatriz Navarro Bravo, José Miguel Latorre, Pablo Fernández-Berrocal

**Affiliations:** ^1^Department of Developmental Psychology and Education, Faculty of Education Science, University of HuelvaHuelva, Spain; ^2^Department of Psychology, Faculty of Medicine, University of Castilla-La ManchaAlbacete, Spain; ^3^Clinical Research Unit of the Integrated Healthcare Office of Albacete, Paraplegics National Hospital FundationAlbacete, Spain; ^4^Applied Cognitive Psychology Unit, Neurological Disabilities Research Institute (IDINE), University of Castilla-La ManchaAlbacete, Spain; ^5^Department of Basic Psychology, Faculty of Psychology, University of MálagaMálaga, Spain

**Keywords:** emotional intelligence, elderly, aging, active reserve, educational level, mediate, moderate

## Abstract

Numerous studies have suggested that educational history, as a proxy measure of active cognitive reserve, protects against age-related cognitive decline and risk of dementia. Whether educational history also protects against age-related decline in emotional intelligence (EI) is unclear. The present study examined ability EI in 310 healthy adults ranging in age from 18 to 76 years using the Mayer–Salovey–Caruso Emotional Intelligence Test (MSCEIT). We found that older people had lower scores than younger people for total EI and for the EI branches of perceiving, facilitating, and understanding emotions, whereas age was not associated with the EI branch of managing emotions. We also found that educational history protects against this age-related EI decline by mediating the relationship between age and EI. In particular, the EI scores of older adults with a university education were higher than those of older adults with primary or secondary education, and similar to those of younger adults of any education level. These findings suggest that the cognitive reserve hypothesis, which states that individual differences in cognitive processes as a function of lifetime intellectual activities explain differential susceptibility to functional impairment in the presence of age-related changes and brain pathology, applies also to EI, and that education can help preserve cognitive-emotional structures during aging.

## Introduction

Cognitive functions decline with age, including the speed of information processing and memory performance (Salthouse et al., 1996; Bisiacchi et al., 2008; Johnson et al., 2009). The severity of age-related cognitive decline varies considerably between individuals, leading researchers to coin the term cognitive reserve (CR) to refer to differences in cognitive function between individuals of the same age (Stern, 2002, 2009). CR has been theorized to comprise passive and active components (Stern, 2009). The passive component reflects pre-existing CR due to brain characteristics such as size or the numbers of neurons and connections. The active component reflects how the brain exploits its cognitive abilities to compensate for aging, such as relying on higher general intelligence or using alternative cognitive strategies. Some cognitive activities are more efficient and resilient to the effects of aging than others (Stern, 2009; Park and Bischof, 2013). Numerous studies have used educational level as a proxy measure of the active component CR. This literature suggests that prior education may protect against age-related cognitive decline and risk of dementia (for a review, Summers et al., 2013). For example, a meta-analysis showed that education reduces the risk of incident dementia (Valenzuela and Sachdev, 2006), while a longitudinal study of Catholic clergy found that education modulates the risk of Alzheimer's disease (Bennett et al., 2003). These findings have been confirmed in the general population (Roe et al., 2007; Santos et al., 2013). In sum, the CR hypothesis states that individual differences in cognitive processes as a function of lifetime intellectual activities explain differential susceptibility to functional impairment in the presence of age-related changes and brain pathology (Barulli and Stern, 2013).

While studies have begun to provide a convincing picture of the ability of education to prevent age-related cognitive decline, few studies have examined the effect of education on age-related decline of emotional intelligence (EI). While researchers agree on the importance of EI for mental health and social and professional functioning, they have conceptualized it in quite different ways. The two most frequent EI models are mixed models and the ability model (Mayer et al., 2008). Mixed models view EI as a conglomeration of characteristics, including empathy, motivation, persistence, optimism, and social skills. Mixed EI is typically measured through self-report instruments, and it overlaps extensively with personality traits and measures of emotional/psychological well-being (Oconnor and Little, 2003; Grubb and McDaniel, 2007; Webb et al., 2013). The ability model, in contrast, defines EI as the integration of several abilities: “the ability to perceive accurately, appraise, and express emotion; the ability to access and/or generate feelings when they facilitate thought; the ability to understand emotion and emotional knowledge; and the ability to regulate emotions to promote emotional and intellectual growth” (Mayer and Salovey, 1997). Ability EI is assessed in adults using the Mayer–Salovey–Caruso Emotional Intelligence Test (MSCEIT; Mayer et al., 2002). MSCEIT is a comprehensive performance test that requires individuals to solve tasks pertaining to each of the four abilities (“branches”) defined by the theory, which are referred to as perceiving, facilitating, understanding, and managing emotions (Mayer et al., 2003). MSCEIT scores show a positive relationship with various domains of daily life, including mental and physical health, social functioning, and academic and workplace performance (e.g., Mayer et al., 2008; Brackett et al., 2011).

Studies of how age affects ability EI have given mixed results. While some studies reported significantly better scores on all four EI branches for older adults (Mayer et al., 1999), other work found age to correlate negatively with emotional perception (Day and Carroll, 2004; Palmer et al., 2005). In fact, a more recent meta-analysis concludes that older people have difficulty recognizing emotions (Ruffman et al., 2008). Still other studies have found no significant association between age and MSCEIT branches (Farrelly and Austin, 2007; Webb et al., 2013). One significant gap in these studies is that they fail to explore a wide range in age; most focus on university populations and enroll participants with an average age younger than 30 years. Thus, studies examining EI in much older individuals are sorely needed.

Even less is known about whether educational level protects against age-related decline in EI. Goldenberg et al. (2006) found educational level to correlate positively with ability EI, but they did not analyze whether EI reserve (equivalent to CR) modulates the relationship between ability EI and age. Specifically, the EI reserve hypothesis is that individual differences in EI as a function of lifetime intellectual activities explain differential vulnerability to functional impairment in the presence of age-related changes. Therefore, the main aim of the present research was to examine educational level and ability EI in older adults from the general population in order to assess whether education can help prevent age-related EI decline. More concretely, our objectives were the following:

- Explore the relationship of ability EI with age and educational level.
- Analyze whether educational level can predict scores for ability EI beyond gender and age.
- Investigate how EI reserve can prevent age-related EI decline, i.e., determine whether educational level mediates and/or moderates the relationship between EI and age.

## Materials and methods

### Participants and procedures

The sample comprised 310 healthy adults (152 men, 158 women) ranging in age from 18 to 76 years (*M* = 42.3, *SD* = 17.2). The self-reported educational level of these participants was classified as primary (60, 19.4%), secondary (64, 20.6%), and university (186, 60%). Participants were recruited through advertisements in adult education centers across Spain and their participation was voluntary. As designated by the advertisement for this research, no compensation, reward, or incentive was offered in exchange for participation in the study. In this investigation, all participants remained for the entire course of the study. They filled out surveys in groups under the supervision of local teachers at the centers.

To participate in our study, volunteers had to be at least 18 years old, they had to be employed or studying at the time of enrollment and they could not have any physical or psychological disability that would compromise their ability to fill out the MSCEIT. Lack of such disability was assessed based on a brief structured interview between participants and local teachers conducted one-on-one. Indeed we focused on volunteers who were students or workers at the time of the study as a way to ensure that they had the minimum cognitive and intellectual abilities to complete the MSCEIT.

### Instruments

*Mayer–Salovey–Caruso Emotional Intelligence Test* (MSCEIT v. 2.0; Mayer et al., 2002; Extremera and Fernández-Berrocal, 2009). Ability EI was measured using a Spanish translation of the MSCEIT that shows similar psychometric properties as the original instrument (Extremera et al., 2006); this test has been validated for adults aged 17 and older. The MSCEIT uses two tasks to measure each of the four branches of EI (perceiving, facilitating, understanding, and managing emotions), comprising a total of eight tasks. The instrument provides separate scores for each branch as well as an overall score for total EI; scores can be calculated based on expert or consensus norms. These two types of norms strongly correlate with each other (*r* > 0.90) (Mayer et al., 2003), and the reliability between the two varies between 0.76 and 0.91 for each of the four branches separately (Mayer et al., 2003). In the present study, we used consensus norms to calculate scores for each of the four branches and for total EI. Scores computed by the test publishers are standardized (*M* = 100, *SD* = 15), and the reliability of the two halves is 0.93 based on the consensus criterion. The test–retest reliability for the global MSCEIT is 0.86 after 3 weeks (Brackett and Mayer, 2003).

### Statistical analysis

All statistical analyses were carried out using the SPSS package (version 20.0; IBM, USA). Preliminary analyses were carried out to compute descriptive statistics, as well as to detect relationships among age, gender, educational level, and ability EI scores. To investigate the validity of age and educational level for predicting ability EI, we conducted three-step hierarchical regression in which gender was entered first (as a control variable), then age, and finally educational level; these variables were correlated with scores for each branch of EI and for total EI. Regression to examine mediation was also performed based on Baron and Kenny's (1986) recommendations, while hierarchical regression to examine moderation was carried out using the PROCESS tool (Hayes, 2013). PROCESS is a macro for moderation and conditional process modeling available for use with SPSS. The PROCESS macro automatically performs the centering and interaction terms and provided the point estimate and first- and second-order variance estimates of the conditional indirect effect at a given set of moderator values.

## Results

### Sample characteristics and correlations among variables

Means, standard deviations, and intercorrelations for the study variables are shown in Table 1. Age correlated negatively with total EI and with three of the four EI branches: perceiving, facilitating, and understanding emotions. It did not show a significant association with managing emotions. Age also correlated negatively with educational level, meaning that older people were less likely than younger ones to have a university education. Gender showed no significant correlation with total EI or with the three branches of perceiving, facilitating, or understanding emotions. In contrast, gender correlated positively with managing emotions, with women showing higher scores than men. Gender did not correlate with educational level. Educational level correlated positively with total EI and with all four EI branches.

**Table 1:** **Means, standard deviations, and intercorrelations among measures**.

	***M***	***SD***	**1**	**2**	**3**	**4**	**5**	**6**	**7**
Gender	0.51	0.50	-						
Age	42.39	17.28	0.18^**^	-					
Educational level	2.41	0.79	−0.03	−0.47^**^	−				
Perceiving emotions	98.37	17.41	0.08	−0.15^**^	0.12^*^	−			
Facilitating emotions	96.55	14.74	0.07	−0.18^**^	0.29^**^	0.52^**^	−		
Understanding emotions	97.24	13.11	−0.01	−0.26^**^	0.36^**^	0.16^**^	0.36^**^	−	
Managing emotions	99.85	17.66	0.17^**^	0.00	0.15^**^	0.23^**^	0.36^**^	0.41^**^	−
Total EI	97.46	13.98	0.11	−0.20^**^	0.30^**^	0.76^**^	0.78^**^	0.62^**^	0.66^**^

* *p < 0.05*,

** *p < 0.01*.

### Analyses of predictive validity

To examine the validity of age and educational level on EI, we conducted three-step hierarchical regression. The independent variables (predictors) were gender, age, and educational level, while the dependent variables were total EI score and scores for each EI branch (perceiving, facilitating, understanding, and managing emotions). We performed the regression by first entering gender in the model, followed by age and finally educational level. We entered gender first because studies of ability EI consistently report significant gender differences (Mayer et al., 2008; Fernández-Berrocal et al., 2012).

Results from the five regression models, corresponding to total EI and to EI scores for the four branches of EI, are shown in Table 2. Gender was not a significant predictor of EI, with the exception of managing emotions, for which women scored significantly higher than men (Δ*R*^2^_MANAGING_ = 0.03). Older age was a significant predictor of lower scores for total EI (Δ*R*^2^ = 0.05), perceiving (0.03), facilitating (0.04), and understanding (0.07). However, age was not a significant predictor of managing emotions. Education level, added last to the models, proved to be a significant predictor of total EI (Δ*R*^2^ = 0.05), facilitating (0.05), understanding (0.07) and managing (0.02), with higher education level predicting higher EI. The effects of educational level on EI over and above the effects of gender and age were medium by Cohen's standards (Cohen, 1988). In addition, including educational level in the model reduced the effects of age on total EI and on the four branches of EI.

**Table 2:** **Hierarchical regression to predict total EI and four EI branches from age and educational level while controlling for gender**.

	**Total EI**	**Perceiving emotions**	**Facilitating emotions**	**Understanding emotions**	**Managing emotions**
	***B***	***SE***	**β**	**Δ*R^2^***	***B***	***SE***	**β**	**Δ*R^2^***	***B***	***SE***	**β**	**Δ*R^2^***	***B***	***SE***	**β**	**Δ*R^2^***	***B***	***SE***	**β**	**Δ*R^2^***
Step 1				0.01				0.01				0.00				0.00				0.03^**^
Gender	3.03	1.58	0.11		2.7	1.98	0.08		2.04	1.67	0.07		−0.26	1.50	−0.01		5.97	1.98	0.17^**^	
Step 2				0.05^**^				0.03^**^				0.04^**^				0.07^**^				0.00
Gender	4.27	1.57	0.15^**^		3.84	1.98	0.11		3.11	1.67	0.10		1.05	1.50	0.04		6.15	2.02	0.17^**^	
Age	−0.18	0.45	−0.23^**^		−0.17	0.06	−0.17^**^		−0.17	0.05	−0.20^**^		−0.20	0.04	−0.27^**^		−0.03	0.06	−0.03	
Step 3				0.05^**^				0.00				0.05^**^				0.07^**^				0.02^**^
Gender	3.88	1.53	0.14^*^		3.73	1.98	0.10		2.70	1.63	0.09		0.62	1.41	0.02		5.81	2.0	0.16^**^	
Age	−0.09	0.05	−0.11		−0.14	0.06	−0.14^*^		−0.06	0.05	−0.07		−0.10	0.05	−0.13^*^		0.06	0.06	0.06	
Educ. level	4.46	1.07	0.25^**^		1.3	1.4	0.06		4.83	1.15	0.26^**^		4.94	1.00	0.30^**^		4.01	1.40	0.18^**^	
Step 4				0.02^**^				0.01^*^				0.03^**^				0.01				0.00
Age^*^ Educ. level	0.25	0.10	0.83^**^		0.25	0.13	0.67^*^		0.33	0.10	1.04^**^		0.17	0.09	0.60		0.01	0.13	0.03	
Total *R*^2^				0.13				0.05				0.12				0.15				0.05

* *p < 0.05*,

** *p < 0.01*.

### Mediation and moderation analyses

Regression showed that including age and educational level simultaneously in the model explained 10% of the variance in total EI when gender was controlled. This finding that age and educational level are related and explain some of the variance in EI led us to construct different mediation and moderation models to test the relationships among these variables. In this way, we investigated the EI reserve hypothesis that educational history can prevent age-related EI decline.

We tested whether educational level mediates the relationship of age with total EI and each of the four EI branches. These mediation analyses were carried out in the steps recommended by Baron and Kenny (1986), and the results are shown in Table 3. Column *a* shows the effect of age on the mediator (educational level); column *b*, the effect of educational level on the dependent variable while controlling age; column *c*, the total effect (direct and indirect) of age on the dependent variable; and column *c*', the direct effect of age on the dependent variable while controlling educational level. The last column shows the results of the Sobel test to assess the statistical significance of the mediation effect. The results show that educational level mediated the relationship of age with total EI and with facilitating and understanding emotions, but not the relationship between age and perceiving emotions. Managing emotions was not included among the dependent variables in the mediation analysis because age did not have a significant direct effect on this variable (see Table 1). Reverse mediation was not significant for any relationship examined.

**Table 3:** Educational level as mediator of the relationship between age and EI^*^.

**Dependent variable**	**Mediator**	***a***	***b***	***c***	**c'**	**Sobel z**
Perceiving emotions	Educ. level	−0.0221^**^ (0.0024)	0.0081 (0.0088)	−0.0011 (0.0004)	−0.0009 (0.0004)	−0.90
Facilitating emotions	Educ. level	−0.0221^**^ (0.0024)	0.0227^**^ (0.0054)	−0.0008^**^ (0.0002)	−0.0003 (0.0003)	−3.82^**^
Understanding emotions	Educ. level	−0.0221^**^ (0.0024)	0.0255^**^ (0.0051)	−0.0011^**^ (0.0002)	−0.0005^*^ (0.0002)	−4.37^**^
Total EI	Educ. level	−0.0221^**^ (0.0024)	0.0181^**^ (0.0044)	−0.0008^**^ (0.0002)	−0.0004 (0.0002)	−3.77^**^

^*^Standard errors are presented in parentheses below the non-standardized B coefficients. Column a shows the coefficient of age in the regression to predict the mediator; column b, the coefficient of the mediator in the regression to predict the dependent variable while controlling age; column c, the coefficient of age in the regression to predict the dependent variable; and column c′, the coefficient of age in the regression to predict the dependent variable while controlling the mediator.

* *p < 0.05*,

** *p < 0.01*.

**Figure 1:**
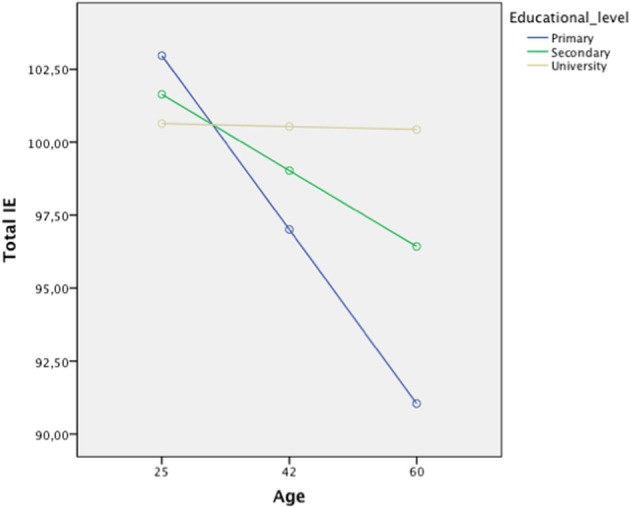
**Assessment of the ability of educational level to moderate the association between age and total EI**.

To examine whether educational level moderates the association of age with total EI and the four EI branches, we performed hierarchical regression using the PROCESS tool (Hayes, 2013). Gender was controlled throughout these analyses and the continuous variables were centered. Educational level was found to moderate the association of age and total EI, perceiving emotions, and facilitating emotions (Table 2, Step 4), while only a marginal effect was observed for understanding emotions (*p* = 0.06). These results suggest that higher educational level predicted higher EI among older adults. In fact, older adults with a university education showed smaller decline in total EI than did older adults with only primary (*t* = −3.09, *p* = 0.002) or secondary education (*t* = −2.94, *p* = 0.003) (Figure 1). Similar results were found for the separate EI branches of perceiving, facilitating, and understanding emotions. Thus, for the sake of parsimony, we depict results based on total EI rather than branches.

## Discussion

Numerous studies have demonstrated that CR protects against age-related decline in cognitive function. To examine whether the equivalent concept of EI reserve protects against age-related decline in EI, we carried out the present study in adults from the general population that were substantially older than the under-30 undergraduates typically examined in the EI research. Since the CR literature routinely uses educational level as a proxy measure of active CR (Bennett et al., 2003; Roe et al., 2007), we used the same variable to represent EI reserve. Specifically, the EI reserve hypothesis is that individual differences in EI as a function of lifetime intellectual activities explain differential vulnerability to functional impairment in the presence of age-related changes. We show that in our population, educational level appears to protect against age-related EI decline, providing the first evidence that educational level helps to counteract gradual losses not only in cognitive function but also in EI.

To examine whether EI indeed declines with age, we measured ability EI in older adults using the MSCEIT. We found that older people scored lower than younger people for total EI and for the three EI branches of perceiving, facilitating, and understanding emotions. In contrast, age was not significantly associated with the EI branch of managing emotions. These findings that age negatively affects numerous aspects of EI are consistent with reviews of clinical and theoretical studies that confirm the inevitability of age-related cognitive decline (e.g., Luszcz, 2011), as well as with a meta-analysis concluding that older people have difficulty recognizing emotions (Ruffman et al., 2008). Our finding that age does not significantly affect emotion management is consistent with behavioral and neuroscience studies associating old age with more stable and satisfying emotional well-being (Carstensen and Mikels, 2005; Scheibe and Carstensen, 2010; Carstensen et al., 2011), and with studies reporting that regulation abilities remain intact in older adults (Lamonica et al., 2010; Ochsner et al., 2012). Therefore, our results suggest that the EI reserve hypothesis applies to three of the four EI branches, but not to the branch of managing emotions, when considered independently of educational level. If educational level is taken into account, then the EI reserve hypothesis may apply even to managing emotions, since we found that people with a university education (who tended to be older) scored higher on this EI branch than did people with a lower educational level.

In fact, we found that the ability EI of adults correlated positively with educational level, and that this variable predicts several dimensions of EI over and above the effects of gender and age, namely, total EI and the EI branches of facilitating, understanding, and managing emotions. These findings are consistent with a previous study showing that educational level improves ability EI in older adults (Goldenberg et al., 2006).

We examined whether educational level mediates and/or moderates the relationship between EI and age. Educational level was found to mediate, and explain some of the age-related variance in, total EI and the EI branches of facilitating and understanding emotions. These findings for EI reserve mirror the results of studies on CR that concluded that education level is associated with better cognitive performance in later life (Gatz et al., 2001; Stern, 2009; Summers et al., 2013). Our moderation analyses showed that higher educational level attenuates age-related EI decline, insofar as older adults with a university education had higher EI scores than older adults with only primary or secondary education. This moderating effect of education has already been reported for cognitive abilities (Bosma et al., 2003). The implication of our work in EI and previous studies with cognitive intelligence is that a higher education level (proxy for EI reserve and CR) increases the likelihood that cognitive-emotional structures remain functional and stable during aging.

Despite the insights from the present work, it has several limitations. Its cross-sectional nature and reliance on a single instrument to assess ability EI precludes inferences about causality in the relationships among age, educational level, and EI. While our mediation analyses support the notion that educational level leads to higher ability EI, prospective studies are needed to address this question rigorously. Our sample was recruited from adult education centers, and the oldest participant was 76 so future studies should seek to include even older individuals. Our study examined exclusively educational level to the detriment of other variables that may help explain the observed variance in cognitive-emotional aging, including socioeconomic status, previous careers, lifestyle engagement, and quality of social relationships. Future studies should take these factors into account to provide a complete picture of the interaction of educational level, EI reserve, and age. Eventually this line of research should be extended to both theory of mind and social cognition (Fernández-Abascal et al., 2013), and EI should be assessed using mixed models or self-reported emotional regulation strategies to complement the performance-based activity EI that we have measured here (Cabello et al., 2013).

Finally, longitudinal interventional studies should be undertaken to test whether EI training can help older adults both with and without university level education against age-related decline in EI and to have a better mental and social life. Such research may provide more insight into how the association between age, EI, and educational level evolves over a lifetime. Evidence suggests that training in social and emotional competencies is crucial and should begin in the first years and be continuous throughout each life-span (Ruiz-Aranda et al., 2012; Rivers et al., 2013).

Our findings contribute to the growing literature on factors that may help protect against age-related decline in cognitive and emotional functioning, which affects quality of life, social adjustment, and professional performance. Educational level has already been shown to protect against age-related cognitive decline by modulating the relationship between age and cognitive abilities (Bennett et al., 2003; Valenzuela and Sachdev, 2006; Roe et al., 2007). Here we show that the same appears to be true for age-related EI decline, at least when older adults with a university education are compared with older adults with a primary or secondary education, or with younger adults of any education level.

### Conflict of interest statement

The authors declare that the research was conducted in the absence of any commercial or financial relationships that could be construed as a potential conflict of interest.
